# (*E*)-3-[(2-Hy­droxy­naphthalen-1-yl)methyl­idene­amino]-5-(morpholin-4-yl­meth­yl)-1,3-oxazolidin-2-one

**DOI:** 10.1107/S1600536811020368

**Published:** 2011-06-18

**Authors:** Na-Na Du, Hua-Jie Xu, Chong-Fu Song, Liang-Quan Sheng

**Affiliations:** a Department of Chemistry, Fuyang Normal College, Fuyang Anhui 236041, People’s Republic of China

## Abstract

The title compound, C_19_H_21_N_3_O_4_, crystallizes with two independent mol­ecules in the asymmetric unit. In both mol­ecules, there is an intra­molecular O—H⋯N hydrogen bond, which correlates with the fact that each mol­ecule adopts an *E* configuration with respect to the C=N bond. In the crystal, there are C—H⋯O and C—H⋯π inter­actions present.

## Related literature

For background to the naphthalene group as a fluoro­phore, see: Li *et al.* (2010 ▶); Iijima *et al.* (2010 ▶). For related structures, see: Xu *et al.* (2009 ▶); Liu *et al.* (2011 ▶). For bond-length data, see: Allen *et al.* (1987 ▶). For hydrogen-bond motifs, see: Bernstein *et al.* (1995 ▶).
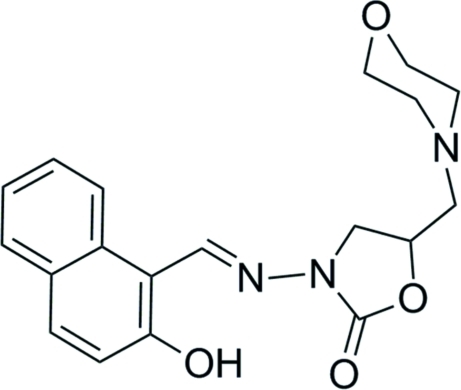


## Experimental

### 

#### Crystal data

C_19_H_21_N_3_O_4_
*M* *_r_* = 355.39
Monoclinic, 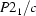

*a* = 10.7764 (6) Å
*b* = 12.0953 (8) Å
*c* = 26.7606 (14) Å
β = 93.452 (5)°
*V* = 3481.7 (4) Å^3^
*Z* = 8
Cu *K*α radiation
μ = 0.79 mm^−1^
*T* = 291 K
0.30 × 0.20 × 0.20 mm

#### Data collection

Oxford Diffraction Xcalibur Sapphire3 Gemini ultra diffractometer
Absorption correction: multi-scan (*CrysAlis PRO*; Oxford Diffraction, 2009 ▶) *T*_min_ = 0.797, *T*_max_ = 0.857
15194 measured reflections
6408 independent reflections
4362 reflections with *I* > 2σ(*I*)
*R*_int_ = 0.025

#### Refinement

*R*[*F* ^2^ > 2σ(*F* ^2^)] = 0.048
*wR*(*F* ^2^) = 0.142
*S* = 1.03
6408 reflections
469 parameters
H-atom parameters constrained
Δρ_max_ = 0.31 e Å^−3^
Δρ_min_ = −0.18 e Å^−3^

### 

Data collection: *CrysAlis PRO* (Oxford Diffraction, 2009 ▶); cell refinement: *CrysAlis PRO*; data reduction: *CrysAlis PRO*; program(s) used to solve structure: *SHELXS97* (Sheldrick, 2008 ▶); program(s) used to refine structure: *SHELXL97* (Sheldrick, 2008 ▶); molecular graphics: *SHELXTL* (Sheldrick, 2008 ▶); software used to prepare material for publication: *SHELXL97*.

## Supplementary Material

Crystal structure: contains datablock(s) global, I. DOI: 10.1107/S1600536811020368/su2276sup1.cif

Structure factors: contains datablock(s) I. DOI: 10.1107/S1600536811020368/su2276Isup2.hkl

Supplementary material file. DOI: 10.1107/S1600536811020368/su2276Isup3.cml

Additional supplementary materials:  crystallographic information; 3D view; checkCIF report

## Figures and Tables

**Table 1:** Hydrogen-bond geometry (Å, °) *Cg*3 is the centroid of the C10–C14/C19 ring.

*D*—H⋯*A*	*D*—H	H⋯*A*	*D*⋯*A*	*D*—H⋯*A*
O4—H4⋯N3	0.82	1.82	2.535 (3)	145
O8—H8⋯N6	0.82	1.84	2.549 (2)	144
C3—H3*B*⋯O2	0.97	2.49	3.141 (3)	124
C9—H9⋯O7	0.93	2.54	3.472 (3)	177
C18—H18⋯O7	0.93	2.57	3.500 (3)	174
C26—H26*A*⋯O3^i^	0.97	2.49	3.403 (3)	157
C36—H36⋯O5^ii^	0.93	2.48	3.371 (3)	161
C23—H23*B*⋯*Cg*3^iii^	0.97	2.65	3.589 (2)	162

Symmetry codes: (i) 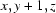; (ii) 
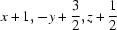
; (iii) 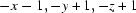.

**Fig. 1.**
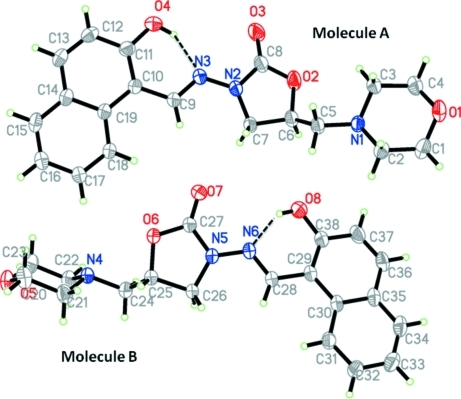
The molecular structure of the title compound, showing the atom numbering scheme and 30% probability displacement ellipsoids.

## supplementary crystallographic information

### Comment

The naphthalene group as a fluorophore has been studied extensively due to its characteristic photophysical properties and the competitive stability in the environment (Li *et al.*, 2010; Iijima *et al.*, 2010). As part of an ongoing study of such compounds based on the naphthalene group (Xu *et al.*, 2009; Liu *et al.*, 2011), we report herein on the crystal structure of the title compound.

The molecular structure of the two independent molecules (A and B) of the title compound is shown in Fig. 1. Both molecules display a *trans* configuration about the C═N bond. The bond distances are within the normal range (Allen *et al.*, 1987). In each molecule there is an intramolecular N-H···O hydrogen bond (Table 1), graph set S(5) (Bernstein *et al.*, 1995), and the oxazolidine rings have chair conformations.

In the crystal molecules are linked via C-H···O and C-H···π interactions (Table 1).

### Experimental

A solution of 3-amino-5-(morpholinomethyl)oxazolidin-2-one (0.10 g, 0.5 mmol) in 5 ml of ethanol was added slowly to a solution of 2-hydro-1- naphthaldehyde (0.086 g,1 mmol) in 10 ml of absolute ethanol under heating and stirring. The mixture was then refluxed for 2 h. The mixture was then cooled to room temperature and the resulting solution was left to stand in air for 15 days. Colourless needle-shaped crystals of the title compound were formed, on slow evaporation of the solvent.

### Refinement

All H-atoms were placed in calculated positions and treated as riding: O—H = 0.82 Å, C—H = 0.93, 0.97 and 0.98 Å, for CH(allyl and aromatic), CH_2_ and CH(methine) H-atoms, respectively, with *U*_iso_(H) = k × *U*_eq_(O,C), where k = 1.5 for OH H-atoms, and k = 1.2 for all other H-atoms.

### Crystal data

**Table:**

C_19_H_21_N_3_O_4_	*F*(000) = 1504
*M**_r_* = 355.39	*D*_x_ = 1.356 Mg m^−^^3^
Monoclinic, *P*2_1_/*c*	Cu *K*α radiation, λ = 1.54184 Å
Hall symbol: -P 2ybc	Cell parameters from 3493 reflections
*a* = 10.7764 (6) Å	θ = 3.3–69.5°
*b* = 12.0953 (8) Å	µ = 0.79 mm^−^^1^
*c* = 26.7606 (14) Å	*T* = 291 K
β = 93.452 (5)°	Needle, colourless
*V* = 3481.7 (4) Å^3^	0.30 × 0.20 × 0.20 mm
*Z* = 8	

### Data collection

**Table:**

Oxford Diffraction Xcalibur Sapphire3 Gemini ultra diffractometer	6408 independent reflections
Radiation source: Enhance Ultra (Cu) X-ray Source	4362 reflections with *I* > 2σ(*I*)
mirror	*R*_int_ = 0.025
Detector resolution: 15.9149 pixels mm^-1^	θ_max_ = 69.6°, θ_min_ = 3.3°
ω scans	*h* = −12→12
Absorption correction: multi-scan (*CrysAlis PRO*; Oxford Diffraction, 2009)	*k* = −9→14
*T*_min_ = 0.797, *T*_max_ = 0.857	*l* = −30→32
15194 measured reflections	

### Refinement

**Table:**

Refinement on *F*^2^	Primary atom site location: structure-invariant direct methods
Least-squares matrix: full	Secondary atom site location: difference Fourier map
*R*[*F*^2^ > 2σ(*F*^2^)] = 0.048	Hydrogen site location: inferred from neighbouring sites
*wR*(*F*^2^) = 0.142	H-atom parameters constrained
*S* = 1.03	*w* = 1/[σ^2^(*F*_o_^2^) + (0.064*P*)^2^ + 0.4406*P*] where *P* = (*F*_o_^2^ + 2*F*_c_^2^)/3
6408 reflections	(Δ/σ)_max_ < 0.001
469 parameters	Δρ_max_ = 0.31 e Å^−^^3^
0 restraints	Δρ_min_ = −0.18 e Å^−^^3^

### Special details

**Table:**

Experimental. CrysAlisPro (Oxford Diffraction, 2009). Empirical absorption correction using spherical harmonics, implemented in SCALE3 ABSPACK scaling algorithm.
Geometry. Bond distances, angles etc. have been calculated using the rounded fractional coordinates. All su's are estimated from the variances of the (full) variance-covariance matrix. The cell esds are taken into account in the estimation of distances, angles and torsion angles
Refinement. Refinement of *F*^2^ against ALL reflections. The weighted *R*-factor *wR* and goodness of fit *S* are based on *F*^2^, conventional *R*-factors *R* are based on *F*, with *F* set to zero for negative *F*^2^. The threshold expression of *F*^2^ > σ(*F*^2^) is used only for calculating *R*-factors(gt) *etc*. and is not relevant to the choice of reflections for refinement. *R*-factors based on *F*^2^ are statistically about twice as large as those based on *F*, and *R*- factors based on ALL data will be even larger.

### Fractional atomic coordinates and isotropic or equivalent isotropic displacement parameters (Å^2^)

**Table:**

	*x*	*y*	*z*	*U*_iso_*/*U*_eq_	
O1	0.62286 (16)	0.34555 (17)	0.81506 (7)	0.0786 (7)	
O2	0.20078 (17)	0.21656 (14)	0.70793 (7)	0.0813 (7)	
O3	0.13641 (18)	0.07867 (14)	0.65635 (8)	0.0913 (8)	
O4	−0.05112 (17)	0.09323 (13)	0.53191 (7)	0.0750 (6)	
N1	0.39342 (15)	0.36978 (14)	0.75659 (6)	0.0505 (6)	
N2	0.08791 (18)	0.26098 (15)	0.63996 (8)	0.0623 (7)	
N3	0.02095 (17)	0.23670 (15)	0.59634 (7)	0.0579 (6)	
C1	0.5528 (2)	0.4447 (2)	0.81593 (10)	0.0772 (10)	
C2	0.4769 (2)	0.4632 (2)	0.76757 (9)	0.0639 (8)	
C3	0.4683 (2)	0.26956 (19)	0.75485 (9)	0.0605 (8)	
C4	0.5440 (2)	0.2549 (2)	0.80360 (10)	0.0746 (9)	
C5	0.31806 (19)	0.38797 (19)	0.71005 (8)	0.0543 (7)	
C6	0.1915 (2)	0.33664 (19)	0.70922 (9)	0.0565 (7)	
C7	0.10901 (19)	0.36726 (18)	0.66250 (8)	0.0532 (7)	
C8	0.1404 (2)	0.1756 (2)	0.66643 (10)	0.0652 (9)	
C9	−0.03316 (19)	0.31476 (18)	0.57062 (8)	0.0511 (7)	
C10	−0.10268 (18)	0.28679 (17)	0.52363 (8)	0.0491 (7)	
C11	−0.1052 (2)	0.17841 (18)	0.50616 (9)	0.0558 (7)	
C12	−0.1633 (2)	0.1521 (2)	0.45919 (9)	0.0615 (8)	
C13	−0.2248 (2)	0.2300 (2)	0.43115 (9)	0.0606 (8)	
C14	−0.23216 (19)	0.34089 (19)	0.44830 (8)	0.0536 (7)	
C15	−0.3021 (2)	0.4208 (2)	0.42112 (9)	0.0653 (8)	
C16	−0.3099 (2)	0.5264 (2)	0.43826 (9)	0.0705 (9)	
C17	−0.2449 (2)	0.5578 (2)	0.48293 (9)	0.0645 (8)	
C18	−0.1759 (2)	0.48169 (18)	0.51017 (8)	0.0566 (7)	
C19	−0.16845 (18)	0.37009 (17)	0.49475 (7)	0.0481 (6)	
O5	−0.49733 (15)	0.85981 (17)	0.44678 (6)	0.0775 (7)	
O6	−0.11211 (15)	0.71887 (13)	0.57561 (6)	0.0656 (6)	
O7	−0.00827 (14)	0.58282 (13)	0.61659 (6)	0.0619 (5)	
O8	0.12904 (18)	0.58845 (14)	0.75372 (7)	0.0786 (7)	
N4	−0.32867 (16)	0.82782 (15)	0.53162 (7)	0.0535 (6)	
N5	−0.01269 (17)	0.76087 (15)	0.64712 (7)	0.0573 (6)	
N6	0.05424 (16)	0.73496 (15)	0.69042 (6)	0.0540 (6)	
C20	−0.3661 (2)	0.8667 (3)	0.44285 (10)	0.0776 (10)	
C21	−0.3015 (2)	0.9028 (2)	0.49156 (9)	0.0677 (9)	
C22	−0.4630 (2)	0.8218 (2)	0.53528 (9)	0.0633 (8)	
C23	−0.5233 (2)	0.7863 (2)	0.48603 (10)	0.0721 (10)	
C24	−0.2674 (2)	0.8597 (2)	0.57977 (9)	0.0602 (8)	
C25	−0.1316 (2)	0.83732 (19)	0.58221 (9)	0.0603 (8)	
C26	−0.0655 (2)	0.86595 (18)	0.63313 (9)	0.0601 (8)	
C27	−0.04061 (19)	0.67765 (18)	0.61369 (8)	0.0519 (7)	
C28	0.09393 (18)	0.81280 (19)	0.71992 (8)	0.0506 (7)	
C29	0.16142 (18)	0.78432 (18)	0.76693 (8)	0.0497 (7)	
C30	0.21472 (18)	0.86931 (19)	0.79892 (7)	0.0492 (7)	
C31	0.2073 (2)	0.9832 (2)	0.78744 (8)	0.0581 (8)	
C32	0.2594 (2)	1.0612 (2)	0.81902 (9)	0.0653 (8)	
C33	0.3214 (2)	1.0305 (3)	0.86422 (10)	0.0759 (10)	
C34	0.3312 (2)	0.9217 (3)	0.87660 (9)	0.0742 (9)	
C35	0.27911 (19)	0.8393 (2)	0.84512 (8)	0.0583 (8)	
C36	0.2897 (2)	0.7256 (2)	0.85764 (9)	0.0703 (9)	
C37	0.2390 (2)	0.6468 (2)	0.82728 (10)	0.0719 (9)	
C38	0.1749 (2)	0.6743 (2)	0.78163 (9)	0.0599 (8)	
H1A	0.60880	0.50670	0.82200	0.0930*	
H1B	0.49770	0.44150	0.84330	0.0930*	
H2A	0.53200	0.47220	0.74050	0.0770*	
H2B	0.42860	0.53040	0.77000	0.0770*	
H3A	0.52330	0.27460	0.72750	0.0730*	
H3B	0.41450	0.20610	0.74890	0.0730*	
H4	−0.02260	0.11530	0.55920	0.1120*	
H4A	0.59390	0.18850	0.80180	0.0900*	
H4B	0.48820	0.24510	0.83040	0.0900*	
H5A	0.36200	0.35820	0.68250	0.0650*	
H5B	0.30880	0.46690	0.70470	0.0650*	
H6	0.15020	0.35910	0.73920	0.0680*	
H7A	0.15170	0.41650	0.64070	0.0640*	
H7B	0.03180	0.40130	0.67130	0.0640*	
H9	−0.02820	0.38760	0.58170	0.0610*	
H12	−0.15950	0.08000	0.44720	0.0740*	
H13	−0.26270	0.21080	0.40020	0.0730*	
H15	−0.34380	0.40140	0.39100	0.0780*	
H16	−0.35850	0.57790	0.42020	0.0850*	
H17	−0.24880	0.63050	0.49400	0.0770*	
H18	−0.13270	0.50380	0.53960	0.0680*	
H8	0.10460	0.61090	0.72590	0.1180*	
H20A	−0.33450	0.79500	0.43350	0.0930*	
H20B	−0.34820	0.91900	0.41680	0.0930*	
H21A	−0.21240	0.90490	0.48810	0.0810*	
H21B	−0.32850	0.97680	0.49980	0.0810*	
H22A	−0.48240	0.76940	0.56110	0.0760*	
H22B	−0.49470	0.89370	0.54440	0.0760*	
H23A	−0.49410	0.71290	0.47800	0.0860*	
H23B	−0.61260	0.78220	0.48880	0.0860*	
H24A	−0.30470	0.81930	0.60630	0.0720*	
H24B	−0.28090	0.93790	0.58530	0.0720*	
H25	−0.09300	0.87820	0.55560	0.0720*	
H26A	−0.00190	0.92170	0.62990	0.0720*	
H26B	−0.12390	0.89100	0.65690	0.0720*	
H28	0.07970	0.88640	0.71130	0.0610*	
H31	0.16600	1.00550	0.75760	0.0700*	
H32	0.25350	1.13560	0.81040	0.0780*	
H33	0.35580	1.08420	0.88570	0.0910*	
H34	0.37330	0.90150	0.90660	0.0890*	
H36	0.33260	0.70500	0.88740	0.0840*	
H37	0.24650	0.57290	0.83670	0.0860*	

### Atomic displacement parameters (Å^2^)

**Table:**

	*U*^11^	*U*^22^	*U*^33^	*U*^12^	*U*^13^	*U*^23^
O1	0.0620 (10)	0.0986 (14)	0.0718 (11)	0.0009 (10)	−0.0250 (9)	0.0030 (10)
O2	0.0908 (12)	0.0537 (10)	0.0932 (13)	−0.0157 (9)	−0.0465 (10)	0.0248 (9)
O3	0.0974 (14)	0.0482 (10)	0.1223 (17)	−0.0019 (9)	−0.0434 (12)	0.0119 (10)
O4	0.0911 (12)	0.0492 (9)	0.0823 (12)	0.0125 (8)	−0.0134 (10)	−0.0102 (9)
N1	0.0469 (9)	0.0519 (10)	0.0513 (10)	−0.0038 (8)	−0.0090 (7)	0.0022 (8)
N2	0.0703 (12)	0.0490 (10)	0.0641 (12)	0.0070 (9)	−0.0241 (10)	0.0024 (9)
N3	0.0594 (11)	0.0526 (10)	0.0593 (11)	0.0059 (9)	−0.0153 (9)	−0.0007 (9)
C1	0.0710 (16)	0.0869 (19)	0.0712 (17)	−0.0147 (15)	−0.0160 (13)	−0.0086 (15)
C2	0.0573 (13)	0.0593 (14)	0.0731 (15)	−0.0080 (11)	−0.0119 (11)	−0.0028 (12)
C3	0.0598 (13)	0.0573 (14)	0.0625 (14)	0.0048 (11)	−0.0112 (11)	0.0029 (11)
C4	0.0751 (16)	0.0746 (17)	0.0713 (16)	0.0082 (14)	−0.0195 (13)	0.0090 (14)
C5	0.0528 (12)	0.0549 (13)	0.0538 (12)	−0.0030 (10)	−0.0090 (10)	0.0088 (10)
C6	0.0524 (12)	0.0575 (13)	0.0579 (13)	−0.0036 (10)	−0.0113 (10)	0.0111 (11)
C7	0.0495 (11)	0.0494 (12)	0.0588 (13)	−0.0014 (9)	−0.0128 (10)	0.0045 (10)
C8	0.0605 (14)	0.0513 (14)	0.0808 (17)	−0.0043 (11)	−0.0214 (12)	0.0129 (12)
C9	0.0509 (11)	0.0476 (11)	0.0535 (12)	0.0022 (9)	−0.0071 (9)	−0.0029 (10)
C10	0.0464 (11)	0.0506 (12)	0.0494 (11)	0.0000 (9)	−0.0038 (9)	−0.0026 (9)
C11	0.0543 (12)	0.0514 (13)	0.0614 (13)	−0.0010 (10)	0.0017 (10)	−0.0078 (11)
C12	0.0632 (14)	0.0580 (13)	0.0634 (14)	−0.0078 (11)	0.0046 (11)	−0.0161 (12)
C13	0.0596 (13)	0.0710 (15)	0.0503 (12)	−0.0147 (12)	−0.0030 (10)	−0.0118 (12)
C14	0.0495 (11)	0.0634 (14)	0.0469 (11)	−0.0076 (10)	−0.0041 (9)	−0.0034 (10)
C15	0.0676 (14)	0.0797 (17)	0.0466 (12)	−0.0090 (13)	−0.0140 (11)	−0.0013 (12)
C16	0.0763 (16)	0.0730 (17)	0.0593 (14)	0.0084 (13)	−0.0191 (12)	0.0062 (13)
C17	0.0757 (15)	0.0584 (14)	0.0570 (13)	0.0094 (12)	−0.0146 (12)	−0.0004 (11)
C18	0.0627 (13)	0.0555 (13)	0.0496 (12)	0.0024 (10)	−0.0141 (10)	−0.0030 (10)
C19	0.0442 (10)	0.0541 (12)	0.0452 (11)	−0.0041 (9)	−0.0026 (8)	−0.0025 (9)
O5	0.0608 (10)	0.1077 (15)	0.0607 (10)	0.0004 (10)	−0.0225 (8)	0.0059 (10)
O6	0.0758 (10)	0.0521 (9)	0.0647 (10)	0.0125 (8)	−0.0309 (8)	−0.0105 (8)
O7	0.0629 (9)	0.0491 (9)	0.0718 (10)	0.0068 (7)	−0.0121 (8)	−0.0026 (8)
O8	0.0984 (13)	0.0559 (10)	0.0779 (12)	0.0015 (9)	−0.0243 (10)	0.0063 (9)
N4	0.0498 (10)	0.0543 (10)	0.0542 (10)	0.0031 (8)	−0.0148 (8)	−0.0011 (9)
N5	0.0673 (11)	0.0489 (10)	0.0526 (10)	0.0057 (9)	−0.0217 (9)	−0.0025 (8)
N6	0.0544 (10)	0.0579 (11)	0.0476 (10)	0.0050 (8)	−0.0136 (8)	0.0009 (9)
C20	0.0683 (16)	0.104 (2)	0.0593 (15)	0.0006 (15)	−0.0062 (12)	0.0150 (15)
C21	0.0546 (13)	0.0737 (16)	0.0728 (16)	−0.0042 (11)	−0.0128 (12)	0.0118 (13)
C22	0.0520 (12)	0.0773 (16)	0.0594 (14)	0.0004 (11)	−0.0074 (10)	−0.0008 (12)
C23	0.0527 (13)	0.095 (2)	0.0670 (16)	−0.0093 (13)	−0.0106 (11)	−0.0060 (15)
C24	0.0588 (13)	0.0608 (14)	0.0587 (13)	0.0054 (11)	−0.0150 (10)	−0.0059 (11)
C25	0.0660 (14)	0.0513 (12)	0.0603 (13)	0.0058 (10)	−0.0224 (11)	−0.0032 (11)
C26	0.0698 (14)	0.0478 (12)	0.0591 (13)	0.0067 (10)	−0.0270 (11)	−0.0040 (10)
C27	0.0479 (11)	0.0505 (13)	0.0558 (12)	0.0018 (9)	−0.0100 (9)	−0.0004 (10)
C28	0.0487 (11)	0.0529 (12)	0.0487 (11)	0.0039 (9)	−0.0090 (9)	0.0015 (10)
C29	0.0446 (11)	0.0576 (12)	0.0457 (11)	0.0036 (9)	−0.0059 (9)	0.0035 (10)
C30	0.0405 (10)	0.0636 (13)	0.0428 (11)	0.0042 (9)	−0.0033 (8)	0.0019 (10)
C31	0.0538 (12)	0.0673 (15)	0.0517 (12)	0.0052 (11)	−0.0085 (10)	−0.0027 (11)
C32	0.0633 (14)	0.0674 (15)	0.0643 (15)	0.0004 (12)	−0.0047 (11)	−0.0108 (12)
C33	0.0713 (16)	0.091 (2)	0.0638 (16)	−0.0079 (15)	−0.0089 (12)	−0.0207 (15)
C34	0.0665 (15)	0.104 (2)	0.0498 (13)	0.0037 (15)	−0.0150 (11)	−0.0031 (14)
C35	0.0482 (12)	0.0807 (16)	0.0449 (11)	0.0046 (11)	−0.0071 (9)	0.0025 (11)
C36	0.0679 (15)	0.0866 (19)	0.0541 (14)	0.0057 (13)	−0.0152 (12)	0.0187 (13)
C37	0.0775 (17)	0.0710 (16)	0.0650 (15)	0.0066 (13)	−0.0133 (13)	0.0204 (13)
C38	0.0595 (13)	0.0600 (14)	0.0586 (13)	0.0044 (11)	−0.0104 (11)	0.0055 (11)

### Geometric parameters (Å, °)

**Table:**

O1—C1	1.418 (3)	C4—H4B	0.9700
O1—C4	1.410 (3)	C4—H4A	0.9700
O2—C6	1.456 (3)	C5—H5A	0.9700
O2—C8	1.347 (3)	C5—H5B	0.9700
O3—C8	1.203 (3)	C6—H6	0.9800
O4—C11	1.352 (3)	C7—H7A	0.9700
O4—H4	0.8200	C7—H7B	0.9700
O5—C23	1.417 (3)	C9—H9	0.9300
O5—C20	1.427 (3)	C12—H12	0.9300
O6—C27	1.337 (3)	C13—H13	0.9300
O6—C25	1.460 (3)	C15—H15	0.9300
O7—C27	1.200 (3)	C16—H16	0.9300
O8—C38	1.355 (3)	C17—H17	0.9300
O8—H8	0.8200	C18—H18	0.9300
N1—C5	1.462 (3)	C20—C21	1.505 (4)
N1—C3	1.459 (3)	C22—C23	1.497 (4)
N1—C2	1.463 (3)	C24—C25	1.486 (3)
N2—C7	1.432 (3)	C25—C26	1.539 (3)
N2—C8	1.356 (3)	C28—C29	1.456 (3)
N2—N3	1.367 (3)	C29—C30	1.435 (3)
N3—C9	1.287 (3)	C29—C38	1.393 (3)
N4—C21	1.448 (3)	C30—C31	1.413 (3)
N4—C24	1.464 (3)	C30—C35	1.428 (3)
N4—C22	1.459 (3)	C31—C32	1.365 (3)
N5—C27	1.368 (3)	C32—C33	1.396 (4)
N5—N6	1.364 (2)	C33—C34	1.360 (5)
N5—C26	1.433 (3)	C34—C35	1.400 (4)
N6—C28	1.285 (3)	C35—C36	1.418 (3)
C1—C2	1.506 (3)	C36—C37	1.347 (3)
C3—C4	1.507 (3)	C37—C38	1.407 (3)
C5—C6	1.498 (3)	C20—H20A	0.9700
C6—C7	1.535 (3)	C20—H20B	0.9700
C9—C10	1.464 (3)	C21—H21A	0.9700
C10—C11	1.392 (3)	C21—H21B	0.9700
C10—C19	1.431 (3)	C22—H22A	0.9700
C11—C12	1.407 (3)	C22—H22B	0.9700
C12—C13	1.353 (3)	C23—H23A	0.9700
C13—C14	1.421 (3)	C23—H23B	0.9700
C14—C19	1.428 (3)	C24—H24A	0.9700
C14—C15	1.402 (3)	C24—H24B	0.9700
C15—C16	1.361 (3)	C25—H25	0.9800
C16—C17	1.401 (3)	C26—H26A	0.9700
C17—C18	1.366 (3)	C26—H26B	0.9700
C18—C19	1.415 (3)	C28—H28	0.9300
C1—H1B	0.9700	C31—H31	0.9300
C1—H1A	0.9700	C32—H32	0.9300
C2—H2B	0.9700	C33—H33	0.9300
C2—H2A	0.9700	C34—H34	0.9300
C3—H3A	0.9700	C36—H36	0.9300
C3—H3B	0.9700	C37—H37	0.9300
			
C1—O1—C4	110.29 (17)	C14—C13—H13	120.00
C6—O2—C8	110.84 (18)	C12—C13—H13	120.00
C11—O4—H4	109.00	C16—C15—H15	120.00
C20—O5—C23	109.40 (19)	C14—C15—H15	120.00
C25—O6—C27	110.75 (17)	C15—C16—H16	120.00
C38—O8—H8	109.00	C17—C16—H16	120.00
C2—N1—C3	108.28 (16)	C16—C17—H17	120.00
C2—N1—C5	110.99 (17)	C18—C17—H17	120.00
C3—N1—C5	112.20 (17)	C17—C18—H18	119.00
N3—N2—C8	117.68 (19)	C19—C18—H18	119.00
C7—N2—C8	114.4 (2)	O5—C20—C21	111.2 (2)
N3—N2—C7	127.94 (18)	N4—C21—C20	110.9 (2)
N2—N3—C9	119.86 (18)	N4—C22—C23	109.58 (19)
C21—N4—C24	112.72 (18)	O5—C23—C22	112.21 (19)
C22—N4—C24	110.55 (17)	N4—C24—C25	112.43 (19)
C21—N4—C22	109.08 (17)	O6—C25—C24	108.78 (18)
N6—N5—C26	127.38 (18)	C24—C25—C26	113.59 (19)
C26—N5—C27	114.42 (18)	O6—C25—C26	105.41 (17)
N6—N5—C27	118.08 (18)	N5—C26—C25	100.79 (17)
N5—N6—C28	119.53 (18)	O6—C27—N5	108.55 (18)
O1—C1—C2	112.0 (2)	O7—C27—N5	127.6 (2)
N1—C2—C1	110.70 (19)	O6—C27—O7	123.9 (2)
N1—C3—C4	109.99 (19)	N6—C28—C29	119.2 (2)
O1—C4—C3	112.6 (2)	C28—C29—C38	120.6 (2)
N1—C5—C6	114.14 (18)	C30—C29—C38	118.99 (19)
O2—C6—C5	110.49 (18)	C28—C29—C30	120.46 (19)
O2—C6—C7	104.98 (18)	C29—C30—C35	119.4 (2)
C5—C6—C7	113.22 (19)	C31—C30—C35	117.03 (19)
N2—C7—C6	101.30 (18)	C29—C30—C31	123.60 (18)
O2—C8—N2	108.4 (2)	C30—C31—C32	121.6 (2)
O3—C8—N2	128.1 (2)	C31—C32—C33	120.7 (2)
O2—C8—O3	123.5 (2)	C32—C33—C34	119.6 (3)
N3—C9—C10	118.80 (19)	C33—C34—C35	121.3 (2)
C9—C10—C11	120.32 (19)	C30—C35—C34	119.8 (2)
C9—C10—C19	120.81 (18)	C34—C35—C36	121.6 (2)
C11—C10—C19	118.86 (19)	C30—C35—C36	118.6 (2)
O4—C11—C10	123.2 (2)	C35—C36—C37	121.3 (2)
O4—C11—C12	116.0 (2)	C36—C37—C38	121.1 (2)
C10—C11—C12	120.8 (2)	O8—C38—C37	116.2 (2)
C11—C12—C13	121.0 (2)	C29—C38—C37	120.6 (2)
C12—C13—C14	120.9 (2)	O8—C38—C29	123.2 (2)
C13—C14—C19	118.8 (2)	O5—C20—H20A	109.00
C15—C14—C19	119.8 (2)	O5—C20—H20B	109.00
C13—C14—C15	121.5 (2)	C21—C20—H20A	109.00
C14—C15—C16	120.9 (2)	C21—C20—H20B	109.00
C15—C16—C17	120.3 (2)	H20A—C20—H20B	108.00
C16—C17—C18	120.0 (2)	N4—C21—H21A	109.00
C17—C18—C19	121.7 (2)	N4—C21—H21B	109.00
C10—C19—C18	123.29 (18)	C20—C21—H21A	109.00
C10—C19—C14	119.50 (19)	C20—C21—H21B	110.00
C14—C19—C18	117.20 (19)	H21A—C21—H21B	108.00
O1—C1—H1A	109.00	N4—C22—H22A	110.00
C2—C1—H1B	109.00	N4—C22—H22B	110.00
H1A—C1—H1B	108.00	C23—C22—H22A	110.00
O1—C1—H1B	109.00	C23—C22—H22B	110.00
C2—C1—H1A	109.00	H22A—C22—H22B	108.00
C1—C2—H2A	109.00	O5—C23—H23A	109.00
N1—C2—H2A	109.00	O5—C23—H23B	109.00
N1—C2—H2B	110.00	C22—C23—H23A	109.00
H2A—C2—H2B	108.00	C22—C23—H23B	109.00
C1—C2—H2B	109.00	H23A—C23—H23B	108.00
C4—C3—H3A	110.00	N4—C24—H24A	109.00
N1—C3—H3B	110.00	N4—C24—H24B	109.00
N1—C3—H3A	110.00	C25—C24—H24A	109.00
C4—C3—H3B	110.00	C25—C24—H24B	109.00
H3A—C3—H3B	108.00	H24A—C24—H24B	108.00
O1—C4—H4A	109.00	O6—C25—H25	110.00
C3—C4—H4A	109.00	C24—C25—H25	110.00
C3—C4—H4B	109.00	C26—C25—H25	110.00
H4A—C4—H4B	108.00	N5—C26—H26A	112.00
O1—C4—H4B	109.00	N5—C26—H26B	112.00
N1—C5—H5B	109.00	C25—C26—H26A	112.00
C6—C5—H5A	109.00	C25—C26—H26B	112.00
N1—C5—H5A	109.00	H26A—C26—H26B	109.00
H5A—C5—H5B	108.00	N6—C28—H28	120.00
C6—C5—H5B	109.00	C29—C28—H28	120.00
C7—C6—H6	109.00	C30—C31—H31	119.00
O2—C6—H6	109.00	C32—C31—H31	119.00
C5—C6—H6	109.00	C31—C32—H32	120.00
N2—C7—H7A	112.00	C33—C32—H32	120.00
N2—C7—H7B	111.00	C32—C33—H33	120.00
C6—C7—H7A	111.00	C34—C33—H33	120.00
C6—C7—H7B	112.00	C33—C34—H34	119.00
H7A—C7—H7B	109.00	C35—C34—H34	119.00
C10—C9—H9	121.00	C35—C36—H36	119.00
N3—C9—H9	121.00	C37—C36—H36	119.00
C11—C12—H12	120.00	C36—C37—H37	119.00
C13—C12—H12	119.00	C38—C37—H37	119.00
			
C4—O1—C1—C2	−55.7 (3)	C9—C10—C11—C12	−174.9 (2)
C1—O1—C4—C3	56.4 (3)	C19—C10—C11—O4	−176.7 (2)
C8—O2—C6—C5	−119.8 (2)	C19—C10—C11—C12	4.7 (3)
C8—O2—C6—C7	2.6 (2)	C9—C10—C19—C14	178.04 (19)
C6—O2—C8—O3	178.7 (2)	C9—C10—C19—C18	−3.0 (3)
C6—O2—C8—N2	−1.5 (3)	O4—C11—C12—C13	177.2 (2)
C23—O5—C20—C21	−57.0 (3)	C10—C11—C12—C13	−4.1 (3)
C20—O5—C23—C22	58.4 (3)	C11—C12—C13—C14	0.2 (3)
C27—O6—C25—C24	−125.1 (2)	C12—C13—C14—C19	2.9 (3)
C25—O6—C27—N5	1.8 (2)	C12—C13—C14—C15	−175.9 (2)
C27—O6—C25—C26	−2.9 (2)	C13—C14—C19—C10	−2.2 (3)
C25—O6—C27—O7	−178.1 (2)	C13—C14—C19—C18	178.82 (19)
C3—N1—C2—C1	−57.3 (2)	C19—C14—C15—C16	0.3 (3)
C2—N1—C3—C4	57.1 (2)	C13—C14—C15—C16	179.1 (2)
C5—N1—C3—C4	179.95 (17)	C15—C14—C19—C10	176.68 (19)
C5—N1—C2—C1	179.14 (17)	C15—C14—C19—C18	−2.4 (3)
C3—N1—C5—C6	90.4 (2)	C14—C15—C16—C17	1.8 (3)
C2—N1—C5—C6	−148.33 (19)	C15—C16—C17—C18	−1.7 (3)
N3—N2—C7—C6	−179.1 (2)	C16—C17—C18—C19	−0.5 (3)
C8—N2—N3—C9	178.3 (2)	C17—C18—C19—C14	2.5 (3)
C7—N2—N3—C9	−0.6 (3)	C17—C18—C19—C10	−176.5 (2)
N3—N2—C8—O3	0.4 (4)	O5—C20—C21—N4	57.7 (3)
C8—N2—C7—C6	2.0 (2)	N4—C22—C23—O5	−59.0 (2)
N3—N2—C8—O2	−179.50 (18)	N4—C24—C25—O6	−60.8 (2)
C7—N2—C8—O2	−0.5 (3)	N4—C24—C25—C26	−177.82 (18)
C7—N2—C8—O3	179.4 (2)	O6—C25—C26—N5	2.7 (2)
N2—N3—C9—C10	179.05 (18)	C24—C25—C26—N5	121.7 (2)
C22—N4—C21—C20	−56.9 (2)	N6—C28—C29—C30	175.43 (19)
C24—N4—C22—C23	−178.56 (19)	N6—C28—C29—C38	−4.8 (3)
C24—N4—C21—C20	179.88 (19)	C28—C29—C30—C31	−0.7 (3)
C21—N4—C22—C23	57.0 (2)	C28—C29—C30—C35	179.22 (18)
C21—N4—C24—C25	−73.4 (2)	C38—C29—C30—C31	179.5 (2)
C22—N4—C24—C25	164.21 (19)	C38—C29—C30—C35	−0.6 (3)
N6—N5—C26—C25	−177.71 (19)	C28—C29—C38—O8	0.9 (3)
C26—N5—N6—C28	−12.1 (3)	C28—C29—C38—C37	−179.4 (2)
C27—N5—N6—C28	172.20 (19)	C30—C29—C38—O8	−179.3 (2)
N6—N5—C27—O7	−3.7 (3)	C30—C29—C38—C37	0.4 (3)
C27—N5—C26—C25	−1.8 (2)	C29—C30—C31—C32	180.0 (2)
N6—N5—C27—O6	176.41 (17)	C35—C30—C31—C32	0.1 (3)
C26—N5—C27—O6	0.1 (3)	C29—C30—C35—C34	−179.9 (2)
C26—N5—C27—O7	−180.0 (2)	C29—C30—C35—C36	0.8 (3)
N5—N6—C28—C29	177.79 (17)	C31—C30—C35—C34	0.0 (3)
O1—C1—C2—N1	57.4 (2)	C31—C30—C35—C36	−179.28 (19)
N1—C3—C4—O1	−58.2 (2)	C30—C31—C32—C33	−0.5 (3)
N1—C5—C6—O2	−68.9 (2)	C31—C32—C33—C34	0.8 (3)
N1—C5—C6—C7	173.73 (18)	C32—C33—C34—C35	−0.7 (3)
C5—C6—C7—N2	118.0 (2)	C33—C34—C35—C30	0.3 (3)
O2—C6—C7—N2	−2.7 (2)	C33—C34—C35—C36	179.6 (2)
N3—C9—C10—C19	177.52 (19)	C30—C35—C36—C37	−0.9 (3)
N3—C9—C10—C11	−2.9 (3)	C34—C35—C36—C37	179.8 (2)
C9—C10—C11—O4	3.7 (3)	C35—C36—C37—C38	0.8 (3)
C11—C10—C19—C14	−1.6 (3)	C36—C37—C38—O8	179.2 (2)
C11—C10—C19—C18	177.4 (2)	C36—C37—C38—C29	−0.6 (3)

### Hydrogen-bond geometry (Å, °)

**Table:**

Cg3, Cg8 and Cg9 are the centroids of the C10–C14/C19, C29/C30/C35–C38 and C30–C35 rings, respectively.

**Table:**

*D*—H···*A*	*D*—H	H···*A*	*D*···*A*	*D*—H···*A*
O4—H4···N3	0.82	1.82	2.535 (3)	145
O8—H8···N6	0.82	1.84	2.549 (2)	144
C3—H3B···O2	0.97	2.49	3.141 (3)	124
C9—H9···O7	0.93	2.54	3.472 (3)	177
C18—H18···O7	0.93	2.57	3.500 (3)	174
C26—H26A···O3^i^	0.97	2.49	3.403 (3)	157
C36—H36···O5^ii^	0.93	2.48	3.371 (3)	161
C2—H2A···Cg9^iii^	0.97	2.99	3.936 (2)	166
C3—H3A···Cg8^iii^	0.97	2.97	3.854 (2)	151
C23—H23B···Cg3^iv^	0.97	2.65	3.589 (2)	162

Symmetry codes: (i) *x*, *y*+1, *z*; (ii) *x*+1, −*y*+3/2, *z*+1/2; (iii) −*x*+1, *y*−1/2, −*z*+3/2; (iv) −*x*−1, −*y*+1, −*z*+1.
